# Quadruple Complication in a Patient With Liver Cirrhosis: A Diagnostic Conundrum

**DOI:** 10.7759/cureus.64953

**Published:** 2024-07-19

**Authors:** Sangita D Kamath, Umesh Kumar, Nilanjan Sarkar, Vikki Shrivastava

**Affiliations:** 1 Internal Medicine, Tata Main Hospital, Jamshedpur, IND; 2 General Medicine, Tata Main Hospital, Jamshedpur, IND; 3 Radiology, Tata Main Hospital, Jamshedpur, IND

**Keywords:** spontaneous, abscess, sepsis, peritonitis, cirrhosis

## Abstract

The incidence of bacterial infections is high in patients with liver cirrhosis (LC) due to compromised immune systems. They are associated with acute hepatic decompensation, multiorgan dysfunction, high morbidity, and mortality and account for 25-46% of all hospitalizations. The mortality rate is about 30% after one month and increases to 63% at one-year follow-up. While spontaneous bacterial peritonitis (SBP), urinary tract infections, soft tissue infections, and respiratory tract infections (pneumonia) are some of the common infections, SBP accounts for 25-31% of the cases and is the most frequent bacterial infection. Impaired activity of the phagocytes of the reticuloendothelial system, decreased production of the complement, and bacteria gaining access into the systemic circulation through the porto-systemic shunts are some of the causes of high-risk bacterial infection in patients with LC. The diagnosis of bacterial infection may be challenging as the typical symptoms like fever may not always be evident. We present a very challenging, middle-aged patient of cirrhosis with diabetes mellitus (DM) who presented with infections at multiple sites like SBP due to Serratia ficaria, multiple pyemic liver abscesses, left peri-nephric abscess with septicaemia, further complicated by portal vein thrombosis - all during single hospital admission. SBP was unique in the sense that no case of SBP due to Serratia ficaria has been published in the literature to date. The stormy clinical course, management, and outcome of the patient are described here.

## Introduction

As per the data published by the WHO in 2017, liver deaths in India account for 2.95% of the total deaths globally [1]. In India, the burden of liver disease and cirrhosis as a cause of mortality has been progressively increasing since 1980 [2]. Some of the complications of cirrhosis contributing to mortality are variceal bleeding, hepatorenal syndrome, hepatic encephalopathy, hepatopulmonary syndrome, hepatic hydrothorax, hepatocellular carcinoma (HCC), SBP, and other bacterial infections. Amongst these, bacterial infections are an important preventable cause of hospitalization in patients with cirrhosis. It is postulated that about 30-60% of patients with liver cirrhosis (LC) will develop bacterial infections [3]. SBP accounts for 25-31% of all infections and is, thus, the commonest bacterial infection in patients with cirrhosis [4,5]. This is due to the poor opsonic activity of the ascitic fluid and is usually caused by gram-negative organisms [5]. We report a rare case of Serratia ficaria associated SBP (which is hitherto not described in the literature) and complicated by multiple pyemic liver abscesses, left perinephric abscesses, and portal vein thrombosis. The patient had a stormy course in the hospital but was ultimately discharged after three weeks.

## Case presentation

A 48-year-old male presented with weakness for one month and swelling in both feet for 15 days duration. He had noticed jaundice 10 days before admission to our hospital. There was a history of passing black tarry stools two to three times a day for the last four to five days and one episode of vomiting coffee-coloured vomitus. He developed a fever with chills two days prior to hospital admission. He had been a chronic alcoholic for the past 10 years, consuming country liquor, about 200 ml per day, most days of the week. He also had a history of type 2 diabetes mellitus for the last five years but was not on regular oral anti-diabetes medications for the same. He had a history of chronic pancreatitis and had undergone cystojejunostomy for pancreatic pseudocyst five years earlier. On examination, he was coherent, had pallor, icterus, bilateral pedal oedema, and was febrile with a pulse rate of 118/minute, low volume, blood pressure of 82/58 mm Hg, respiratory rate of 22/minute with accessories working and oxygen saturation on ambient air (SpO2) of 98%. Examination of the gastrointestinal system revealed ascites with mild splenomegaly (two fingers below the left costal margin). There were a few dilated veins over the abdominal wall. The rest of the systemic examination was within normal limits. His initial and subsequent blood reports during the hospital stay were as in Table 1. Ultrasound of the abdomen showed a slightly enlarged liver with increased echotexture, mild splenomegaly, and moderate ascites.

**Table 1:** Lab investigations (haematological and biochemical tests) ESR: Erythrocyte sedimentation rate

Investigations	Day 1	Day 5	Day 11	Day 16	Day 24
Haemoglobin (gm/dl)	6.2	7.1	9.7	7.8	9.8
Total leucocyte (cu mm)	15,380	8,060	6,510	5,340	4,890
Differential count (%)	N-79 % L- 18%	N –75% L -19%	N – 69% L – 20%	N–68% L– 22%	N– 69% L – 22%
Mean Corpuscular Volume (fl)	83.6	85.6	84.0	89.8	90.1
Platelet count (cu mm)	79,000	84,000	86,000	1,06,000	1,08,000
Reticulocyte count (%)	4.5	3.4	-	-	3.1
Serum lactate dehydrogenase (LDH) (N:140-280 IU/L)	533.7	465	-	-	235.3
Total serum proteins (g/dl)	5.2	4.9	5.1	4.9	5.6
Serum albumin (g/dl)	2.14	2.05	2.03	2.01	2.37
Serum globulin (g/dl)	3.06	2.85	2.98	2.89	3.23
Serum creatinine (mg/dl)	0.6	0.57	0.52	0.68	0.52
Total bilirubin (mg/dl)	3.95	2.99	3.6	2.63	1.68
Direct bilirubin (mg/dl)	2.2	1.86	1.8	1.34	0.8
Alanine transaminase (ALT) (U/L)	44.1	20.9	24.5	25.2	17.2
Aspartate transaminase (AST) (U/L)	94.2	86.9	49.2	39.2	35.3
Alkaline Phosphatase (ALP) (U/L)	234.1	302.7	423.5	412.5	206.3
Prothrombin time (INR)	1.82	2.08	1.75	1.48	1.13
ESR (Westergren) (mm1st hour)	76	69	72		34
Serum lipase	35		32		
C-Reactive Protein (mg/dl)	12.4	13.1	4.3	3.3	2.1
Procalcitonin (mg/dl)	5.2	3.62	2.4		1.6

**Figure 1:**
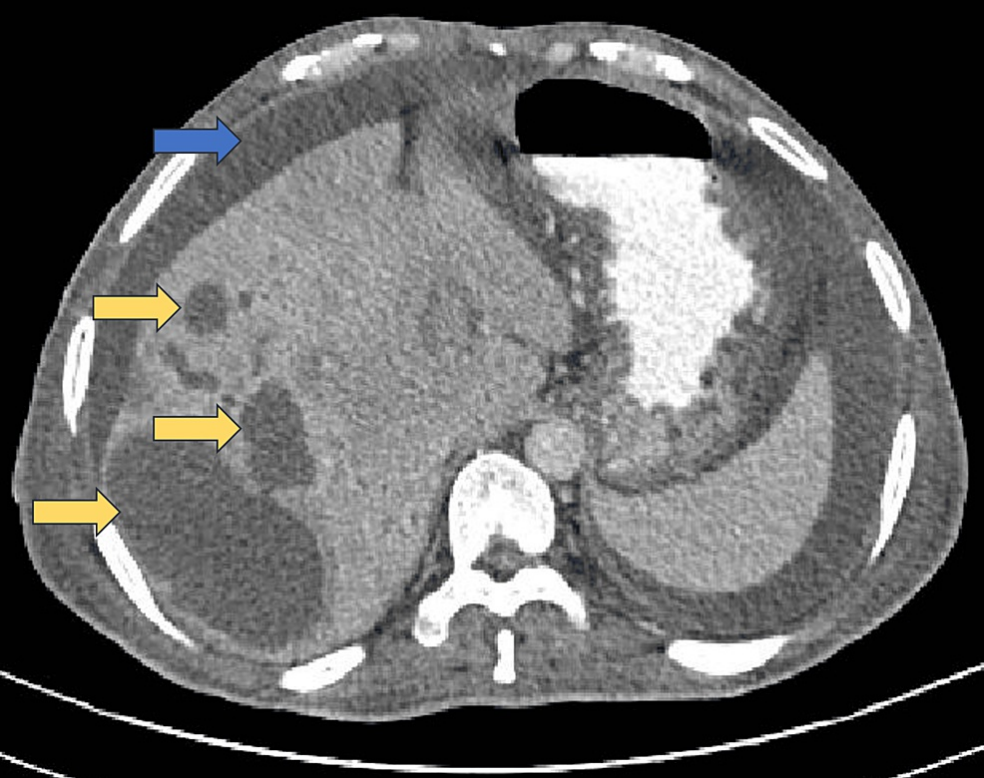
CECT abdomen (axial section) CECT: Contrast-enhanced computerised tomography The image shows multiple abscesses (yellow arrows) and ascites (blue arrow) in the right lobe of the liver.

**Figure 2:**
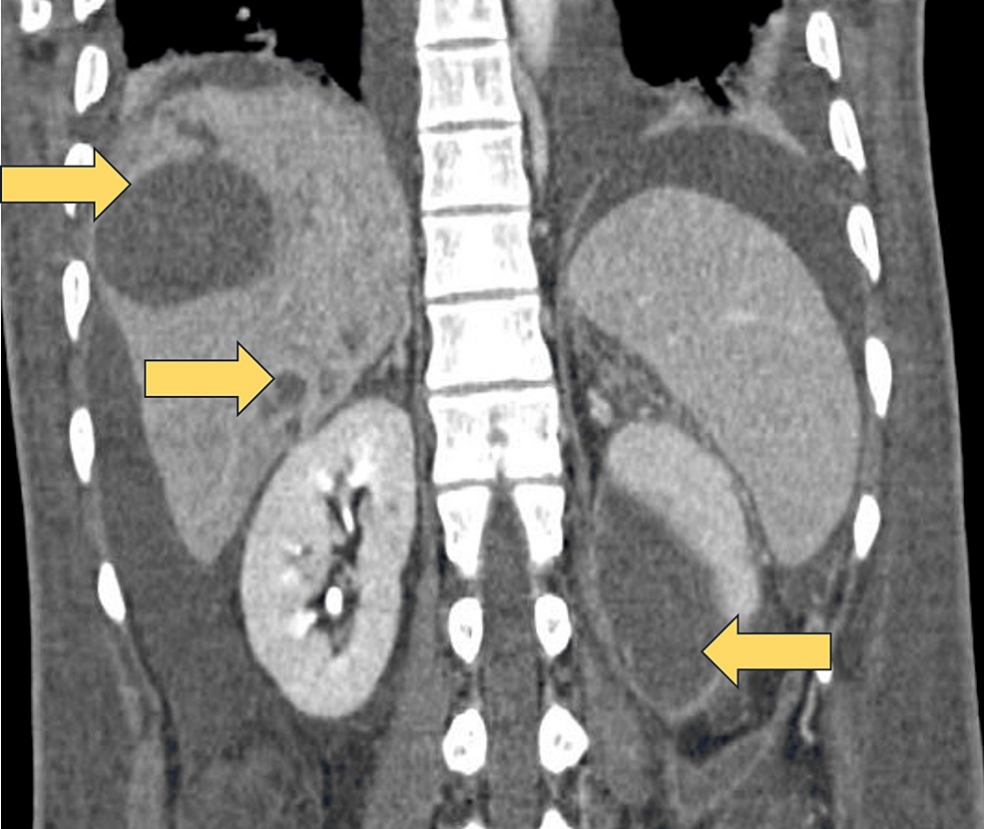
CECT abdomen (coronal section) CECT: Contrast-enhanced computerised tomography The image shows multiple abscesses in the liver and left perinephric abscess (yellow arrows).

**Figure 3:**
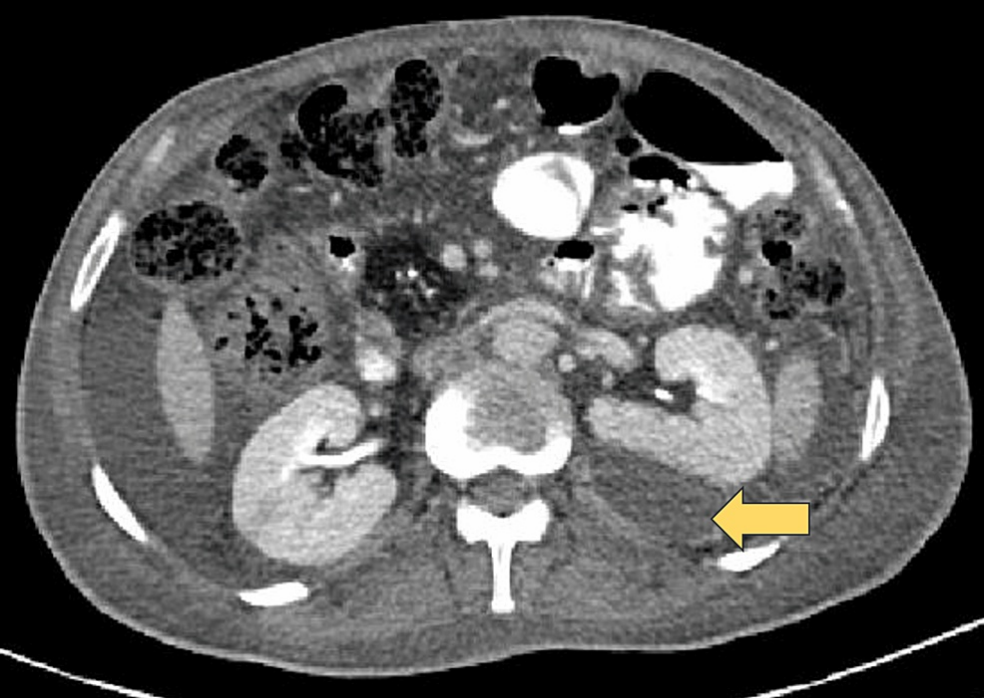
CECT abdomen (axial section) CECT: Contrast-enhanced computerised tomography The image shows a left perinephric abscess (yellow arrow).

Further, ascitic fluid analysis showed total 1,920 cells with 90% neutrophils, 5% lymphocytes, 5% reactive mesothelial cells (Table 2), glucose-193 mg/dl, lactose dehydrogenase (LDH)-185.4 U/dl, proteins -.4 g/dl, albumin-0.62 g/d, adenosine deaminase (ADA) level of 4.4 U/L indicative of transudate ascites with neutrophilic predominance. Smear revealed plenty of neutrophils, few lymphocytes and reactive mesothelial cells suggestive of class ll benign inflammatory pathology. Malignant cells were not found. Cultures for fungi were negative. The culture of ascitic fluid grew *Serratia ficaria,* which was sensitive to meropenem, amikacin, and tigecycline. Blood culture (BactT/ALERT 3D) grew Gram-negative organism *Escherichia coli* (*E. coli*), which was sensitive to carbepenams, piperacillin-tazobactam and tigecycline (minimum inhibitory concentration, MIC<0.5). Contrast-enhanced computerised tomography (CECT) of the abdomen showed enlarged liver, irregular capsular outline with multiple hypoechoic areas in both lobes, right more than the left with caudate lobe hypertrophy, volume loss of the head and uncinate process of the pancreas, moderate ascites, splenomegaly and left perinephric collection. CECT abdomen was, thus, suggestive of multiple, pyemic liver abscesses with decompensated liver disease, left perinephric abscess, and chronic pancreatitis (Figures 1-3*)*.

Upper gastrointestinal endoscopy (UGIE) showed three columns of grade 2 oesophageal varices and mild portal hypertensive gastropathy (PHG). Ultrasound guided aspiration from the hypoechoic areas was done, histopathology of which revealed sheets of neutrophils suggestive of abscess. There were no malignant cells. A smear of aspirated material showed chains and clusters of cocci while culture grew gram-negative rods - *E. coli* (sensitivity pattern as mentioned above) and gram-positive cocci - *Enterococcus faecalis* sensitive to tigecycline (MIC<0.12), teicoplanin (MIC<0.5), vancomycin (MIV=1) and linezolid (MIC=2). Non-contrast magnetic resonance imaging (MRI) of the abdomen showed an enlarged liver with an irregular margin and multiple ill-defined, varied-sized lesions in both lobes, more on the right, hypodense on T1, and hyperintense on T2/FS with central necrosis and air foci. One of the abscesses was large and measured 9.2 cm x 7.4 cm in segment seven of the liver. The portal vein was dilated and the right and left intrahepatic branches showed thrombus within. There was subcapsular renal collection (measuring 9.2 x 3.4 cm) at the lower pole of the left kidney. Mild ascites and left pleural effusion were also noted. Chest radiography revealed obliteration of both costophrenic angles. A colonoscopy done to rule out malignancy, was normal. Magnetic Resonance Cholangio-Pancreatography (MRCP) did not show obstruction to intra/extrahepatic biliary or pancreatic ducts. The patient was empirically treated with intravenous (IV) piperacillin-tazobactam 4.5 gm thrice daily and metronidazole 500 mg thrice daily, which was later changed to meropenem 1 gm thrice daily and tigecycline 100 mg bolus followed by 50 mg twice daily based on the antibiogram report. A pig-tail catheter drainage of the largest abscess was attempted but failed as the abscess was not liquified. During the initial part of the hospital stay, the patient was in septic shock and was managed with inotropic support with noradrenaline (maximum dose 5 mcg/minute) for five days. Later, he also had a worsening of ascites, which required therapeutic tapping up to 3 litres on three occasions with intravenous 20% human albumin supplementation. The patient gradually improved clinically and became afebrile. Ascitic fluid cell count repeated after 96 hours of initiating antibiotics showed a decrease in the total cell count to 960 cells/cu mm with 75% neutrophils *(*Table 2*)*. Intravenous antibiotics were continued for 24 days.

On day 25 of the hospital stay, the patient was discharged on oral antibiotics (oral faropenem 300 mg twice daily and oral clindamycin 600 mg twice daily) and asked to follow up in the out-patient department after two weeks with a plan to drain the abscess with pig-tail catheter if liquified or surgical drainage if catheter drainage is not feasible. Ascitic fluid cell count repeated before discharge showed a further decrease in the total cell count to 90 cells/cu mm with 10% neutrophils and 75% lymphocytes (Table 2).

**Table 2:** Comparative analysis of ascitic fluid cell count WBCs: White blood cells

Parameters	Day 1	Day 4	Day 15	Day 22
Total WBCs	1,920	960	440	90
Neutrophils	90%	75%	25%	10%
Lymphocytes	5%	20%	65%	75%
Others	5%	5%	10%	15%

A final diagnosis of ethanol-related decompensated liver disease with portal hypertension, oesophageal varices, pyemic liver abscesses with septic portal vein thrombosis, spontaneous bacterial peritonitis (SBP) with septic shock in the background of chronic pancreatitis and type 2 diabetes mellitus (T2DM) was made.

## Discussion

Our patient had alcohol-related decompensated chronic liver disease (CLD) - cirrhosis with pyemic liver abscesses, SBP, left perinephric abscess, and septicemia in the background of chronic pancreatitis. Patients of CLD have reduced host defence against bacteria with impaired bacterial clearance. Further, gut bacterial overgrowth (gut dysbiosis) and increased gut permeability facilitate bacterial translocation, extra-intestinal dissemination, and development of SBP [5]. It occurs in 7-30% of hospitalised patients with liver cirrhosis (LC) and in 1 to 3.5% of the out-patients of cirrhosis [3]. SBP is defined as an infection of ascitic fluid characterised by positive ascitic fluid culture and/or fluid absolute polymorphonuclear cell count ≥ 250/cu mm in the absence of a surgically treatable cause [3,4]. Some of the risk factors for the development of SBP include low ascitic fluid (AF) total protein (<1.5 g/dl), severe hepatic impairment as suggested by Model for End-stage Liver Disease (MELD) and Child-Turcotte-Pugh (CTP) scores, hyponatremia (130 mmol/L), acute variceal bleed and use of proton-pump inhibitor [4,5]. Our patient had an upper gastrointestinal bleed with hyponatremia, increasing the risk for SBP.

The bacterial aetiology of SBP includes Gram-negative bacilli (GNB), Gram-positive cocci (GPC), multidrug-resistant (MDR) organisms, and anaerobes, with GBN being the most causative organisms [5,6]. Over the years, there has been a changing paradigm in the bacteriology of SBP. GPC was found to be the predominant group (56.1%) isolated in an observational study done in France [7] and also in hospitalised patients who develop SBP (nosocomial SBP). In a cross-sectional study done by Nguyen et al. from July 2019 to July 2020, the gram-negative organisms isolated were *E. coli* (53%), *Klebsiella pneumoniae* (6%), *Burkholderia cepacia* (11%) and *Aeromonas veronii* (6%) [8]. In our case, the organism isolated was *Serratia ficaria*, which is a Gram-negative facultative anaerobe that belongs to the family Yersiniaceae. It was first described by Grimont et al. in 1979 as a component of the fig tree ecosystem [9]. It is found in figs and fig pollinators, like wasps and is an opportunistic pathogen in humans. In a comprehensive literature review on *Serratia ficaria* infections over the past 40 years, Lucchi et al. described 18 human infections, which included gall bladder empyema, respiratory tract infection, endophthalmitis, septicaemia and skin injury lesions. There were reports from those countries where fresh fig consumption was high (like US, Greece, Turkey etc) [10]. Literature search to date did not reveal this organism as a cause of SBP. Also, no case of *Serratia ficaria* infection has been reported from India. Our patient denied the consumption of fresh figs in the recent past, and also, there were no fig trees in the vicinity of his house. It was intriguing as to how our patient acquired this infection. The initial treatment of SBP includes broad-spectrum antibiotics (third-generation cephalosporins) with albumin supplementation [5,6]. Antibiotics are then tailored as per the drug sensitivity pattern of the organism.

Our patient also had multiple pyemic liver abscesses from which both Gram-positive and Gram-negative organisms (Enterococcus and *E.coli*) were grown. *E. coli *was also cultured from the blood and the perinephric abscess. Cultures could be negative in 30% of the cases if samples are drawn after starting the antibiotics [11]. Though 80% of the liver abscesses are bacterial in origin in the United States, amoebic liver abscess (ALA) is still the predominant cause in India, observed in 60% of the cases, and is caused by the extra-intestinal spread of Entamoeba to the liver [12]. ALAs are usually solitary and found in the right lobe of the liver. However, in 15% of the cases, ALA may be multiple like most bacterial (pyemic) abscesses. Premathilake et al. described a liver abscess caused by *Klebsiella pneumoniae*, which was single, solid, and located in the right lobe, mimicking ALA [13]. Between 50 to 60% of the pyemic abscesses originate in the biliary tract [12]. Other sources include appendicitis, malignancy with super-added infection, diverticulitis and post-surgical infection. Rarely, it could arise from dissemination during bacteraemia, as in our case. There would have been seeding of the liver and perinephric space during the bacteraemia phase. Pyemic abscess are often polymicrobial in nature as in our case.

Risk factors for developing pyogenic liver abscess are diabetes mellitus, malnutrition, immunosuppression, and advanced age. Diagnosis is made based on radiological grounds and culture. Treatment of choice includes third generation cephalosporins initially and later, the drugs are tailored as per the antibiogram of the isolate. The duration of the treatment is two to six weeks, depending upon the response. Pig-tail catheter drainage is considered if the abscess is more than 5 cm and or involves the left lobe of the liver [12]. It can be left in situ for four to six weeks and is removed when the output is less than 10 ml in a day. Rarely, surgical drainage is indicated for multiloculated abscesses and abscesses with thick, purulent material that cannot be drained freely [14]. Early use of effective antimicrobial therapy has resulted in a decline in the mortality rate from 24% in earlier series to 3% in recent studies [12]. Rupture of the liver abscess into the peritoneal cavity with the development of secondary bacterial peritonitis seems to be unlikely in our case, as the organisms isolated from ascitic fluid and liver abscess were different and there was no ultrasound evidence of rupture.

Our patient also had portal vein thrombosis (PVT). This could be related to the liver cirrhosis or could be septic PVT (pylephlebitis), due to liver abscess. As there was no portal cavernoma suggesting chronic thrombosis, PVT was acute, probably due to liver abscess. Syed et al. reported PVT in 24% of 64 cases of liver abscess in a five-year study [15], while the estimated annual prevalence of PVT in patients with cirrhosis is 0.6-15.8% [16]. Treatment of septic PVT includes antibiotics. However, the role of anticoagulation in the setting of liver disease is controversial as the risk of bleeding remains high [16].

We, thus, report this case of liver cirrhosis with a conundrum of complications as described. To the best of our knowledge, such a case has not been reported earlier in the literature. The idea of this presentation is to make clinicians aware of such rare presentations. Failure to recognise concurrent infections can result in a delay in the initiation of proper treatment, resulting in high morbidity and mortality.

## Conclusions

Though bacterial infections are reported in patients with liver cirrhosis, the development of multiple concurrent infections is extremely rare. In such cases, the selection of antibiotic therapy should be guided by the sensitivity pattern of the organisms and the severity of the infection. Timely diagnosis and appropriate management are required for a successful outcome.
